# Influence of Interannual Climate Variability on Wheat Quality During Long-Term Storage: Comparative Analysis and Mathematical Modeling

**DOI:** 10.3390/foods15152726

**Published:** 2026-08-03

**Authors:** Diana Petronela Poetelea, Emilian Mosnegutu, Claudia Tomozei, Narcis Barsan, Eniko Gaspar, Diana Mirila, Mihail Balan, Grzegorz Przydatek

**Affiliations:** 1 Department of Environmental Engineering, Mechanical Engineering and Agritourism, Faculty of Engineering, “Vasile Alecsandri” University of Bacau, 600115 Bacau, Romania; axintediana90@gmail.com (D.P.P.); claudia.tomozei@ub.ro (C.T.); narcis.barsan@ub.ro (N.B.); 2 Faculty of Agricultural Sciences, Food Industry and Environmental Protection, “Lucian Blaga” University of Sibiu, 550024 Sibiu, Romania; eniko.gaspar@ulbsibiu.ro; 3 Faculty of Mechanical Engineering and Transport, Technical University of Moldova, MD-2004 Chisinau, Moldova; mihail.balan@pmai.utm.md; 4 Department of Engineering Science, University of Applied Sciences in Nowy Sącz, Zamenhofa 1a, 33-300 Nowy Sącz, Poland; g.przydatek@gmail.com

**Keywords:** wheat storage, climate variability, grain quality, mathematical modeling, quality degradation

## Abstract

This study investigates the influence of interannual climate variability on wheat quality during long-term storage under identical technological conditions. The same wheat cultivar was stored during two consecutive periods (2023–2024 and 2024–2025) in a flat warehouse without mechanical aeration or forced cooling, while environmental parameters (temperature and relative humidity) and quality indicators (grain moisture content, test weight, gluten content, and protein content) were continuously monitored. Based on the experimental data, empirical predictive models describing the relationships between storage time, environmental conditions, and wheat quality indicators were developed using nonlinear regression and curve-fitting techniques implemented in TableCurve 3D. Two model categories were established: a transfer model for moisture and temperature evolution and a degradation model for test weight, gluten, and protein changes. The models showed good predictive performance, with coefficients of determination ranging from R^2^ = 0.82 to 0.97. Model performance was assessed using residual analysis and statistical performance indicators, yielding low prediction errors and high Nash–Sutcliffe efficiency coefficients (0.95–0.99). The 2024–2025 period exhibited slightly greater thermal variability, with atmospheric thermal amplitude increasing from approximately 33 °C to 34 °C and grain thermal amplitude increasing from approximately 30 °C to 32 °C compared with the 2023–2024 storage period. This was accompanied by differences in moisture evolution patterns and more pronounced reductions in test weight, gluten, and protein content. These findings suggest that climate variability is associated with changes in hygrothermal conditions and wheat quality deterioration processes, while the proposed models provide reliable tools for predicting quality evolution and supporting adaptive grain storage management under changing climatic conditions.

## 1. Introduction

The quality of stored agricultural products, particularly wheat (*Triticum aestivum* L.), is a key factor in maintaining their nutritional, technological, and commercial value during long-term storage [1,2]. In recent years, increasing climate variability, characterized by rising temperatures, fluctuations in relative humidity, and the growing frequency of extreme weather events, has emerged as a significant challenge for post-harvest grain management [3,4,5,6]. These climatic changes can affect storage stability and accelerate quality degradation processes, making the control of storage conditions a critical component of modern agri-food supply chains [3,4]. Effective storage management is therefore essential for minimizing post-harvest losses, preserving grain quality, and ensuring food security [4,5,7,8]. Climatic factors directly influence the physical, chemical, and biological processes occurring within stored grain bulk, thereby affecting the stability and preservation of wheat quality during long-term storage [9,10]. Temperature and relative humidity are considered the most critical environmental parameters because they regulate grain respiration, moisture migration, metabolic activity, and microbial growth [11,12,13,14]. Elevated temperatures and excessive humidity levels can accelerate quality deterioration by promoting fungal development, increasing respiration rates, and reducing important technological characteristics such as protein content, gluten strength, and test weight [15]. Recent studies have demonstrated that wheat stored under controlled temperature and moisture conditions maintains significantly better processing and storage quality than grain exposed to fluctuating environmental conditions [4,8,11].

Maintaining appropriate grain moisture content and storage temperature is essential for preserving wheat quality throughout extended storage periods [16,17]. Several recent studies have demonstrated that storage environment significantly influences the evolution of biochemical and technological quality parameters in wheat grains [18,19,20]. Long-term storage under elevated temperatures has been associated with changes in enzyme activity, protein characteristics, wet gluten content, and falling number values, whereas lower storage temperatures contribute to better quality preservation [21,22]. Furthermore, investigations conducted under different storage environments have shown that fluctuations in temperature and moisture may affect wheat quality evolution and accelerate deterioration processes, highlighting the importance of continuous monitoring and optimized storage management practices [23,24]. In addition to physicochemical deterioration, inappropriate storage conditions may promote fungal growth and increase the risk of mycotoxin contamination, thereby affecting both grain quality and food safety [9,25,26,27]. Consequently, effective environmental control and continuous monitoring are essential to minimize contamination risks and ensure the long-term safety and marketability of stored grain [25,28]. The adoption of IoT-based monitoring platforms has further improved the capability to track storage conditions and support early intervention strategies aimed at maintaining grain quality [29,30,31].

Recent studies have highlighted the increasing influence of climatic variability on the preservation of wheat quality during storage [32,33]. Variations in environmental temperature and humidity affect grain moisture dynamics, biochemical activity, fungal development, and the evolution of key technological quality parameters [32]. In addition to climatic factors, storage-induced quality changes are influenced by the storage system employed and the biological status of the grain mass [33]. Improved storage technologies, innovative monitoring approaches, and predictive models have been shown to contribute to better preservation of grain quality and reduced post-harvest losses during extended storage periods [33,34,35,36]. Despite these advances, comparative studies evaluating consecutive storage periods under identical storage conditions remain limited, highlighting the need for further investigations into the direct impact of climatic variability on long-term wheat storage performance [32,33,34]. Recent advances in intelligent storage systems and predictive analytics have enabled more accurate forecasting of quality changes in stored wheat by integrating environmental monitoring, machine learning techniques, and decision-support algorithms [37,38].

Under the storage conditions investigated in the present study, quality changes were expected to be primarily associated with moisture migration within the grain bulk and temperature-dependent biochemical processes occurring during storage. Although microbial activity and fungal development are recognized as potential contributors to grain deterioration, these mechanisms were not directly evaluated through microbiological analyses in the current work. The observed changes in test weight, gluten content, and protein content should therefore be interpreted mainly as responses to the combined effects of environmental temperature, relative humidity, and the resulting moisture equilibration processes occurring within the stored grain mass. Consequently, the present study focuses on the relationships between environmental conditions and quality evolution rather than on the direct identification of specific microbiological or biochemical degradation pathways. Therefore, the present study proposes a comparative approach by analyzing the same wheat variety stored under identical storage conditions during two consecutive storage periods (2023–2024 and 2024–2025). This approach provides an opportunity to examine the extent to which differences in climatic conditions between two consecutive storage years may be associated with changes in wheat quality evolution under similar technological conditions. Particular attention is given to the relationships between environmental conditions, grain moisture, protein content, wet gluten, and test weight, which are among the most important indicators of wheat quality. By comparing the behavior of the stored grain under similar management practices but different climatic scenarios, the study aims to provide valuable information for optimizing long-term storage strategies and improving grain preservation under changing climatic conditions. It should be noted that the two analyzed periods correspond to consecutive storage years of the same grain lot rather than to two independent harvest lots.

The findings of this research are expected to contribute to a better understanding of the mechanisms governing quality changes in stored wheat and to support the development of adaptive storage management practices [39]. Furthermore, the results may assist grain storage operators in identifying critical periods associated with quality deterioration and in implementing preventive measures aimed at reducing post-harvest losses, maintaining technological quality, and improving food security within cereal supply chains. Based on these considerations, the present study was designed to investigate the relationship between climatic variability and wheat quality evolution during long-term storage. The main objective of this study is to comparatively evaluate the influence of climatic factors on wheat quality during long-term storage under identical storage conditions across two consecutive years.

The specific objectives were: (i) to analyze the relationships between climatic parameters and the physicochemical characteristics of wheat; (ii) to identify interannual differences in the evolution of quality parameters; (iii) to evaluate the association between climatic variability and wheat quality deterioration processes; (iv) to evaluate the evolution of the main wheat quality parameters during storage; and (v) to develop recommendations for adaptive grain storage strategies under changing climatic conditions.

It should be acknowledged that the present study was conducted using a single wheat cultivar stored in one commercial storage facility over two consecutive storage periods. Although the experimental design maintained identical storage technology, monitoring procedures, and management conditions across both years, the observed differences cannot be attributed exclusively to climatic variability with complete certainty. Therefore, the results should be interpreted as evidence of climate-associated effects under the specific storage conditions investigated rather than as definitive proof of causal climatic relationships. The primary objective of the present work was the development and validation of empirical predictive models based on experimental observations collected during two consecutive storage periods. Consequently, the study focused on model identification and predictive performance rather than on formal statistical correlation analyses among the investigated variables.

## 2. Materials and Methods

Common wheat (*Triticum aestivum* L.), cultivar RO 1, compliant with SR 13548/2013 specifications, harvested in 2023, was used in the present study. Prior to storage, the grain was cleaned and conditioned according to the standard operational procedures applied within the storage facility. The initial physicochemical characteristics of the wheat, determined immediately after conditioning and before storage, are presented in Table 1.

The study had a comparative design, investigating the evolution of wheat quality parameters during two consecutive storage periods (2023–2024 and 2024–2025) under similar technological conditions. The main objective was to assess the influence of interannual climatic variability on the stability of the physicochemical characteristics of wheat during long-term storage.

The study was conducted on a single commercial wheat lot (cultivar RO1) harvested in 2023 and introduced into storage immediately after post-harvest conditioning. The same grain lot was monitored continuously throughout the entire study period. For comparative purposes, the monitoring data were subsequently grouped into two consecutive storage intervals corresponding to the first year of storage (2023–2024) and the second year of storage (2024–2025). Consequently, the physicochemical characteristics presented in Table 1 represent the initial condition of the grain at the beginning of the storage process and serve as the common baseline for both monitoring intervals.

Storage time was treated as a continuous variable throughout the two-year monitoring period. The distinction between the 2023–2024 and 2024–2025 intervals was introduced only for comparative analysis and does not represent a reset of the storage process or the introduction of a new grain lot.

The evaluated quality parameters included grain moisture content, grain temperature, test weight, wet gluten content, and protein content. The selected quality parameters were chosen because they are routinely used in commercial wheat storage and quality grading systems and are directly related to the technological and market value of the grain. Although starch is an important wheat constituent and may undergo structural modifications during storage, its overall content generally exhibits smaller quantitative changes under conventional storage conditions than protein- and gluten-related parameters. Therefore, the present study focused on quality indicators considered more sensitive to climatic variability and more relevant for practical grain storage management. These parameters were selected due to their importance in assessing the technological quality of wheat and their sensitivity to environmental and storage conditions. Monitoring was carried out over a period of 12 months for each storage cycle, from September to August of the following year, covering the periods 2023–2024 and 2024–2025.

Approximately 500,000 kg of wheat was monitored throughout the study period. The grain was stored in a flat warehouse designed for long-term cereal storage. The product was packed in bags with a capacity of approximately 1000 kg and arranged under uniform storage conditions throughout both experimental periods in order to minimize the influence of technological factors on the obtained results. The monitored wheat consisted of a single storage lot of approximately 500,000 kg introduced into the warehouse at the beginning of the storage period. Although the grain was packed in bags of approximately 1000 kg capacity, these bags were not treated as independent experimental units because they formed part of the same storage lot and were maintained under identical storage conditions. Throughout the study, representative composite samples were obtained from multiple locations within the stored grain mass and used for quality analysis and model development.

The storage system ensured a constant configuration of the grain bulk throughout the entire monitoring period. Temperature and humidity measurements within the stored grain were performed using vertically installed probes placed at representative locations inside the storage area. A total of 10 sensors were installed inside the storage facility for continuous measurement of temperature and relative humidity. The sensors were distributed throughout the storage area and positioned at intervals corresponding to approximately every five grain bags in order to obtain representative information regarding the spatial distribution of environmental conditions within the stored grain mass. The vertical arrangement of the sensors allowed the assessment of temperature and moisture distribution throughout the grain bulk and facilitated the identification of possible differences between central and peripheral storage zones.

Ventilation of the storage facility was achieved exclusively through natural air circulation, without the use of forced aeration or cooling systems. No technological interventions, such as grain turning or redistribution, were performed during the monitoring periods, allowing the natural behavior of the stored product to be evaluated under static storage conditions.

Furthermore, no additional insecticidal or fungicidal treatments were applied to the analyzed grain lots during storage in order to avoid artificially influencing the evolution of quality parameters and to accurately assess the effects of storage microclimate conditions on wheat quality.

Sampling was performed systematically throughout the entire storage period. More than 50 elementary samples were collected from different locations within the stored grain bulk and combined to obtain a representative composite sample for each determination. Sampling was conducted four times per month, on the 1st, 10th, 20th, and 30th day of each month, resulting in a total of 48 determinations for each experimental year. Each composite sample was subsequently analyzed in triplicate, and the values presented in the figures represent the arithmetic mean of the three analytical determinations.

Repeated measurements collected throughout the storage period were treated as a chronological time-series dataset describing the evolution of the monitored parameters over time. The primary objective was to characterize temporal trends and develop empirical predictive models rather than to perform inferential statistical comparisons between individual sampling dates.

The physicochemical parameters were determined using standardized analytical methods and metrologically calibrated equipment. Grain moisture content was measured using a Granomat grain moisture analyzer (Pfeuffer GmbH, Kitzingen, Germany, 2015; measurement range 5–40% moisture), while protein content was determined using a Granolyser HL grain analyzer (Pfeuffer GmbH, Kitzingen, Germany, 2017) based on near-infrared spectroscopy (NIR). Both instruments were periodically calibrated and verified according to the manufacturer’s recommendations throughout the experimental period. Wet gluten content was determined according to SR EN ISO 21415-2:2015 [40], while test weight was determined in accordance with SR EN ISO 7971-3:2019 [41] using the standard bulk density method.

At the beginning of the storage period, the following environmental parameters were recorded: storage air temperature of 24 °C, storage relative humidity of 45%, outdoor temperature of 25 °C, and outdoor relative humidity of 40%.

The experimental study was carried out in a storage facility located in the contact area between Neamț and Bacău counties under similar technological conditions during both monitoring periods. Environmental monitoring was performed using a digital LoRaWAN-based system that enabled the continuous recording of temperature and relative humidity both within the grain bulk and in the surrounding environment.

The monitoring system installed in the storage facility consisted of an SMT-LR1 platform equipped with a WAP-LR8 LoRaWAN communication unit and an application server based on a DELL OPTIPLEX 300 MT computer. The storage facility was equipped with SST-LR1 probes for temperature monitoring and TUT-LR1 probes for simultaneous temperature and humidity monitoring, all featuring local display and LoRaWAN wireless data transmission capabilities.

Environmental parameters were recorded automatically at predefined monitoring intervals. All data were electronically stored and centralized for subsequent analysis. To ensure measurement accuracy and reproducibility, the sensors were periodically verified and calibrated in accordance with the applicable metrological requirements and technical specifications for long-term grain storage monitoring systems.

The complete time-series dataset used for graphical representation and model development is provided as Supplementary Table S1.

## 3. Results

Following the analysis of the mean values obtained from triplicate determinations of the physicochemical characteristics of the wheat samples during the 2023–2024 and 2024–2025 storage periods, a series of graphical representations were developed to identify and compare the relationships among the analyzed parameters.

**Figure 1:**
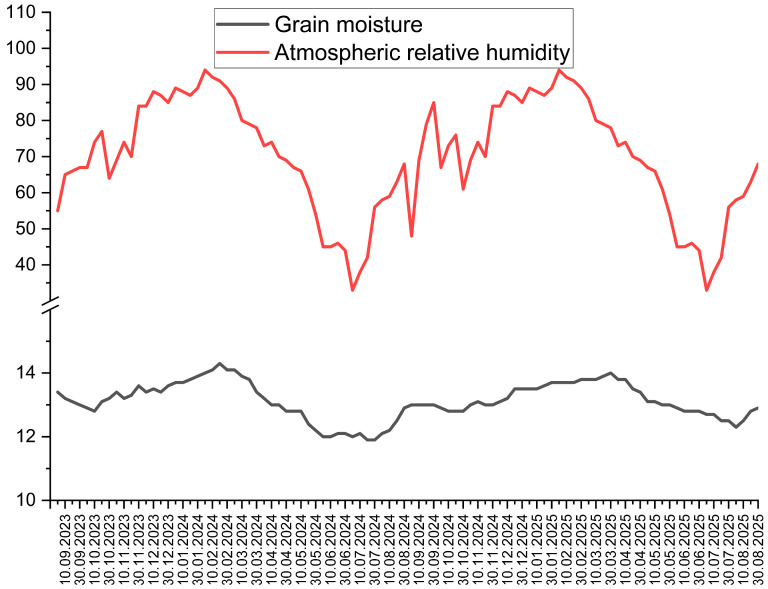
Comparative variation of atmospheric relative humidity and grain moisture content during the 2023–2024 and 2024–2025 storage periods.

The variation in atmospheric relative humidity and grain moisture content throughout the monitoring periods (2023–2024 and 2024–2025) is presented in Figure 1. Grain moisture values represent the mean of three analytical replicates obtained for each sampling date.

Atmospheric relative humidity exhibited very similar behavior during the two storage periods. In both years, the minimum recorded value was 33%, while the maximum reached 94%. The average values recorded over the monitoring periods were 69.77% in 2023–2024 and 70.25% in 2024–2025. The small difference observed between these averages (0.48 percentage points) indicates a high degree of interannual stability in atmospheric relative humidity under the investigated conditions.

A similar analysis performed for grain moisture content revealed slight differences between the two storage cycles. During the 2023–2024 period, moisture content varied between 11.9% and 14.3%, with an average value of 13.06%. In the 2024–2025 period, the minimum and maximum values were 12.3% and 14.0%, respectively, while the average moisture content increased to 13.17%, representing a difference of 0.11 percentage points compared with the previous year.

Overall, atmospheric relative humidity remained highly stable between the two storage periods, whereas grain moisture content displayed more noticeable differences between the two storage periods. The 2024–2025 period was characterized by a minimum moisture content that was 0.4 percentage points higher, a maximum moisture content that was 0.3 percentage points lower, and a moisture variation range that was 0.7 percentage points narrower than those observed during the 2023–2024 period.

**Figure 2:**
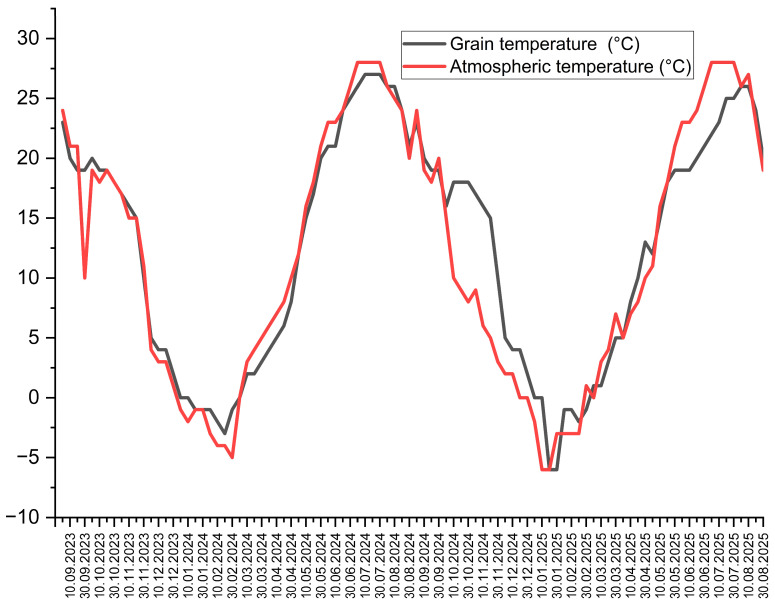
Comparative variation of atmospheric temperature and average grain temperature during the 2023–2024 and 2024–2025 storage periods.

The variation in atmospheric temperature and grain temperature during the monitoring periods (2023–2024 and 2024–2025) is presented in Figure 2.

In the graphical representation, the upper section illustrates the variation in ambient temperature within the storage environment, while the lower section shows the variation in the temperature of the stored grain. Analysis of the obtained results highlights several important observations.

Regarding atmospheric temperature, the 2023–2024 period was characterized by a minimum value of approximately −5 °C and a maximum value of approximately 28 °C, resulting in a thermal amplitude of about 33 °C. During the 2024–2025 period, the minimum and maximum temperatures were approximately −6 °C and 28 °C, respectively, corresponding to a thermal amplitude of about 34 °C. Comparison of the two periods indicates an increase in thermal amplitude of approximately 1 °C, representing a rise of about 3.03%.

The variation in the average grain temperature exhibited a similar trend but with lower fluctuations. During the 2023–2024 period, the minimum grain temperature was approximately −3 °C, while the maximum reached approximately 27 °C, resulting in a thermal amplitude of about 30 °C. In the 2024–2025 period, grain temperature varied between approximately −6 °C and 26 °C, corresponding to a thermal amplitude of about 32 °C. Consequently, the thermal amplitude increased by approximately 2 °C, equivalent to an increase of about 6.67% compared with the previous storage period.

A comparison between atmospheric temperature and grain temperature reveals that the stored grain mass experienced lower temperature fluctuations than the surrounding environment. This observation is consistent with the buffering effect commonly reported for bulk-stored grain, although the present study did not directly quantify thermal inertia or response lag. In both monitoring periods, the thermal amplitude of the grain remained lower than that of the storage environment by approximately 3 °C during 2023–2024 and 2 °C during 2024–2025, indicating a dampened thermal response of the stored product.

Overall, atmospheric temperature exhibited more pronounced seasonal fluctuations directly influenced by external climatic conditions, whereas grain temperature showed comparatively lower variability and a reduced thermal amplitude. These observations support the existence of a buffering effect of the stored grain mass relative to ambient conditions, although the magnitude of any temperature-response delay was not quantified in the present study. Furthermore, the 2024–2025 storage period was characterized by a slight increase in thermal variability, reflected by an increase in thermal amplitude from 33 °C to 34 °C for the storage environment and from 30 °C to 32 °C for the stored grain mass.

**Figure 3:**
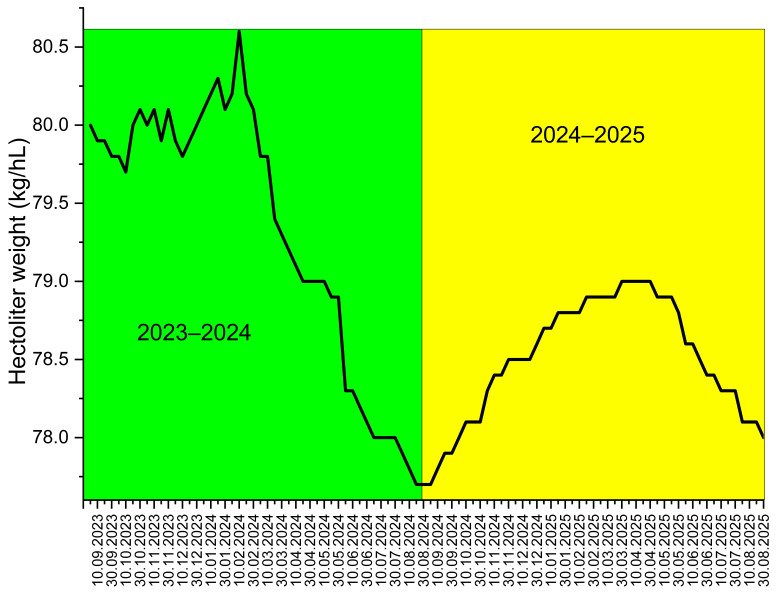
Comparative variation of the test weight of stored wheat during the 2023–2024 and 2024–2025 storage periods.

The variation in the test weight of the stored wheat during the 2023–2024 and 2024–2025 storage periods is presented in Figure 3. Analysis of the graphical results highlights several important trends regarding the evolution of this quality parameter during long-term storage.

During the 2023–2024 storage period, test weight ranged from a maximum value of 80.1 kg/hL to a minimum value of 78.4 kg/hL, resulting in a variation amplitude of 1.7 kg/hL. The average value recorded throughout the monitoring period was approximately 79.2 kg/hL.

For the 2024–2025 period, the maximum recorded test weight was 80.0 kg/hL, while the minimum value decreased to 77.8 kg/hL, resulting in a variation amplitude of 2.2 kg/hL. The average test weight during this period was approximately 78.9 kg/hL.

A comparison of the two storage periods indicates a reduction in the average test weight of approximately 0.3 kg/hL, corresponding to a decrease of about 0.38%. At the same time, the variation amplitude increased by approximately 0.5 kg/hL during the 2024–2025 period, representing an increase of about 29.41%.

Overall, test weight exhibited a gradual declining trend throughout storage. Although this behavior has frequently been associated in the literature with respiration-related dry matter losses and structural changes occurring during storage, these mechanisms were not directly evaluated in the present study. Therefore, the observed decline is reported as an empirical observation associated with storage duration and environmental conditions.

**Figure 4:**
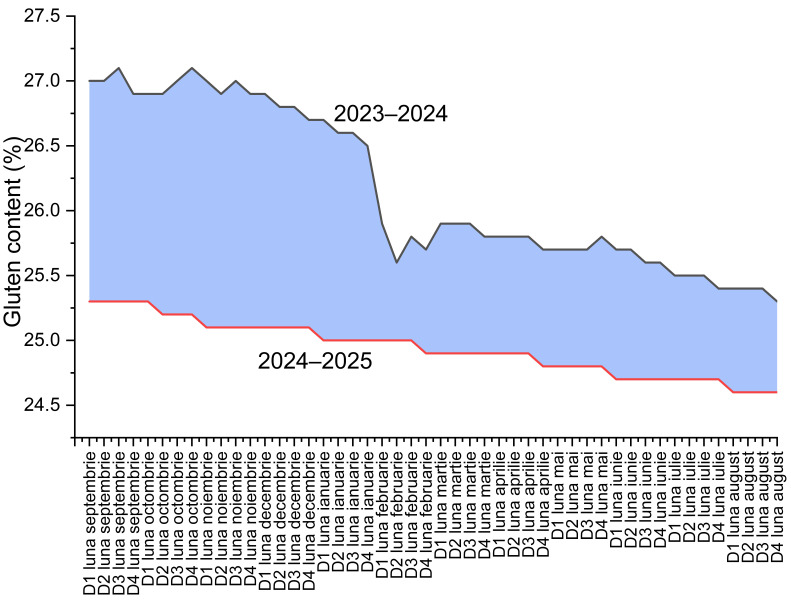
Comparative variation of the wet gluten content of stored wheat during the 2023–2024 and 2024–2025 storage periods.

The variation in the wet gluten content of the stored wheat during the 2023–2024 and 2024–2025 storage periods is presented in Figure 4. Analysis of the graphical results reveals several important aspects regarding the evolution of this key technological quality parameter during long-term storage.

During the 2023–2024 storage period, wet gluten content ranged from a maximum value of 27.1% to a minimum value of 25.8%, resulting in a variation amplitude of 1.3 percentage points. The average gluten content recorded throughout the monitoring period was approximately 26.4%.

For the 2024–2025 storage period, the maximum wet gluten content reached 27.0%, while the minimum value decreased to 25.1%, resulting in a variation amplitude of 1.9 percentage points. The average value recorded during this period was approximately 26.0%.

A comparison of the two storage periods indicates a reduction in the average gluten content of approximately 0.4 percentage points, corresponding to a decrease of about 1.52%. Furthermore, the variation amplitude increased by approximately 0.6 percentage points during the 2024–2025 period, representing an increase of about 46.15%.

Overall, wet gluten content exhibited a more pronounced decline during the 2024–2025 storage period. The differences observed between the two storage cycles may be associated with differences in environmental conditions experienced during storage. However, because no formal statistical significance testing was performed and both storage periods had the same duration, the relative contributions of climatic variability and storage-related deterioration cannot be distinguished with certainty. Nevertheless, the recorded values remained within acceptable limits for wheat intended for bread-making applications.

**Figure 5:**
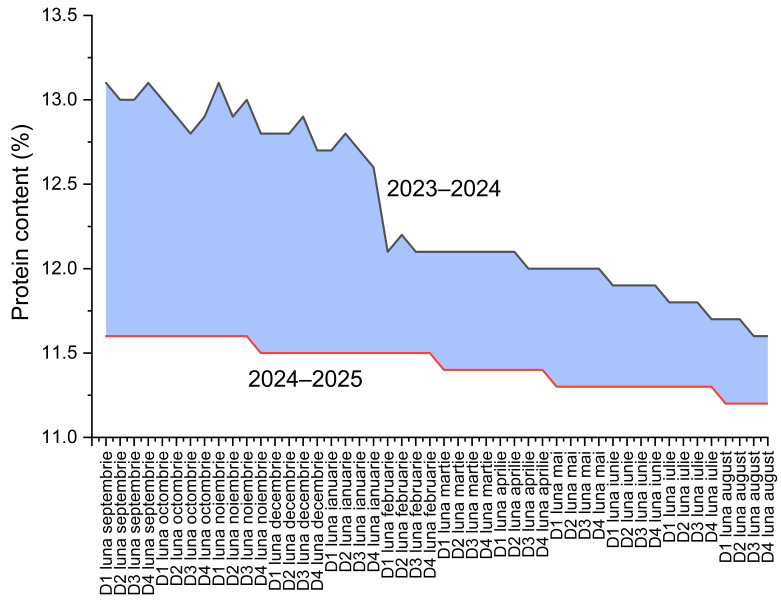
Comparative variation of the protein content of stored wheat during the 2023–2024 and 2024–2025 storage periods.

The variation in the protein content of the stored wheat during the 2023–2024 and 2024–2025 storage periods is presented in Figure 5. Analysis of the graphical results provides important information regarding the evolution of this technological quality parameter during long-term storage.

During the 2023–2024 storage period, protein content varied between a maximum value of 13.1% and a minimum value of 12.5%, resulting in a variation amplitude of 0.6 percentage points. The average protein content recorded throughout the monitoring period was approximately 12.8%.

For the 2024–2025 storage period, the maximum protein content remained 13.1%, while the minimum value decreased to 12.2%, resulting in a variation amplitude of 0.9 percentage points. The average value recorded during this period was approximately 12.6%.

A comparison of the two storage periods reveals a decrease in the average protein content of approximately 0.2 percentage points, corresponding to a reduction of about 1.56%. In addition, the variation amplitude increased by approximately 0.3 percentage points during the 2024–2025 period, representing an increase of about 50%.

Overall, protein content exhibited a gradual declining trend throughout storage, a phenomenon associated with the biochemical changes occurring within the grain mass during long-term preservation. The obtained results suggest that climatic conditions and storage duration may both contribute to changes in the technological quality of wheat during storage. However, the present experimental design does not allow their individual effects to be quantified separately.

## 4. Mathematical Model Identification

Nonlinear regression and curve-fitting techniques for mathematical model identification represent fundamental tools for the analysis of complex phenomena in engineering and applied sciences [42,43]. The applicability of these approaches has been demonstrated in numerous fields, including mechanical engineering, transport systems, structural analysis, and process optimization [44,45,46,47,48,49]. Although these studies address phenomena different from grain storage, they highlight the ability of nonlinear regression methods and TableCurve 3D-based model identification procedures to describe complex systems characterized by multiple interacting variables. Consequently, the present work applies the same mathematical framework to the study of wheat storage processes and quality evolution under varying climatic conditions.

Unlike most statistical and regression software packages, where the user must define in advance the type of equation to be fitted (e.g., linear, polynomial, exponential, logarithmic, or trigonometric), TableCurve 3D contains an extensive equation library and automatically evaluates a large number of candidate mathematical relationships for the same experimental dataset [50,51,52]. This feature enables the identification of equations that provide the best compromise between statistical accuracy, mathematical complexity, and interpretability of the results.

The objective of the modeling procedure was not to derive mechanistic equations from fundamental heat and mass transfer principles, but rather to develop empirical predictive models capable of reproducing the observed evolution of the monitored parameters under the investigated storage conditions [45,46,47,48,49,53,54]. Model selection was based on the coefficient of determination (R^2^), physical relevance of the identified relationships, and ease of implementation in practical applications.

**Figure 6:**
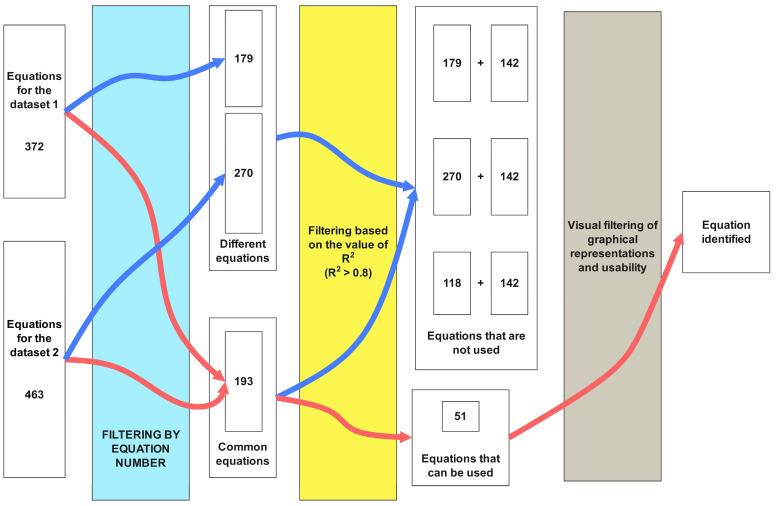
Methodological framework for selecting the mathematical equation describing mass and energy transfer processes during grain storage.

**Figure 7:**
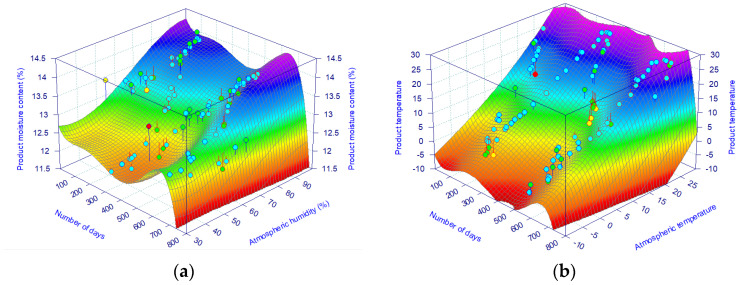
Three-dimensional response surfaces obtained for: (**a**) Dataset 1; (**b**) Dataset 2.

**Figure 8:**
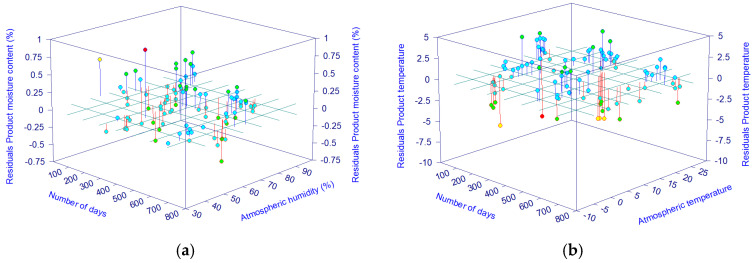
Residual plots for: (**a**) Dataset 1; (**b**) Dataset 2.

**Figure 9:**
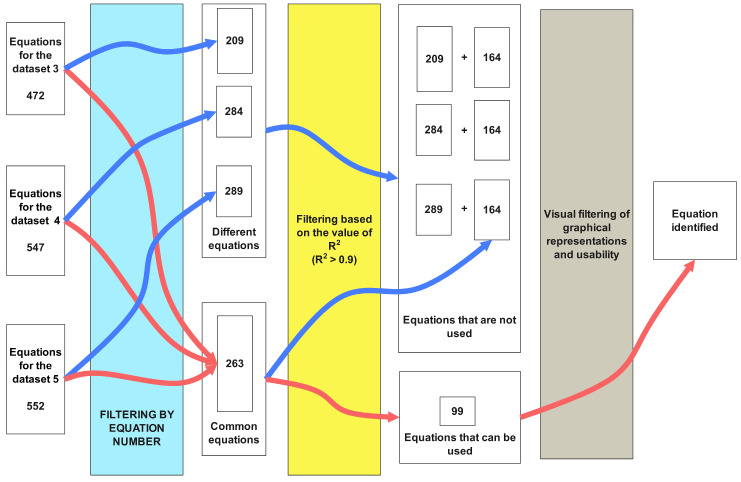
Workflow for the Selection of the Mathematical Equation Describing Quality Parameter Degradation Processes during Storage.

The equation-selection procedure was not based solely on the highest coefficient of determination (R^2^). TableCurve 3D (v.4.0) was initially employed to generate a comprehensive library of candidate equations applicable to the investigated datasets. Subsequently, a structured multistage filtering procedure was implemented in Microsoft Excel, as illustrated in Figure 6, Figure 7, Figure 8 and Figure 9. Candidate equations were screened according to predefined performance thresholds, coefficient consistency, numerical stability, and applicability across multiple monitored parameters. Preference was given to equation families capable of providing satisfactory predictive performance for several quality indicators simultaneously rather than to equations offering marginally higher R^2^ values for individual datasets. This procedure reduced the number of candidate models and favored mathematical formulations that provided an appropriate balance between predictive accuracy, mathematical consistency, and practical applicability.

The adopted modeling strategy was intended to develop empirical predictive relationships suitable for practical grain-storage applications rather than mechanistic models derived from fundamental transport or reaction-kinetics equations. Consequently, the objective was to identify mathematical functions capable of reproducing the observed behavior of the monitored parameters under real storage conditions while maintaining a reasonable balance between predictive accuracy, mathematical simplicity, and practical applicability.

Accordingly, the selected equations should be interpreted as empirical predictive models obtained from experimental observations rather than mechanistic formulations derived from first-principles representations of heat transfer, mass transfer, or biochemical degradation processes.

To evaluate model predictive capability, a temporal validation strategy was adopted. Experimental data collected during the 2023–2024 storage period were used for model calibration, whereas data collected during the 2024–2025 storage period were reserved for temporal validation. Because both datasets originated from the same grain lot monitored continuously throughout storage, this procedure should be interpreted as temporal validation rather than independent external validation. Model performance was subsequently assessed using MSE, RMSE, MAE, MAPE, and Nash–Sutcliffe efficiency (NSE) indicators.

Because both calibration and validation datasets originated from observations collected from the same grain lot during a continuous storage process, the validation dataset cannot be considered an independent experimental population. Consequently, the reported validation results should be interpreted as evidence of predictive consistency within the investigated dataset rather than as independent external verification.

For the identification of three-dimensional mathematical models, the experimental datasets were organized according to the nature of the analyzed variables and the parameters of interest monitored during the storage process.

Since time represents one of the essential parameters of the study and was initially expressed as specific calendar days corresponding to the monitoring period, such a notation cannot be directly employed in the mathematical modeling procedure. Therefore, the temporal variable was reformulated by expressing it as the elapsed period from the introduction of the product into storage, namely the storage time.

This data organization enabled both a clear distinction between the physical and biochemical phenomena under investigation and a systematic approach to the mathematical modeling process. Accordingly, the following datasets were defined:

• Dataset 1: storage time (day), atmospheric relative humidity, and product moisture content;
• Dataset 2: storage time (day), atmospheric temperature, and product temperature;
• Dataset 3: storage time (day), atmospheric temperature, and test weight;
• Dataset 4: storage time (day), atmospheric temperature, and gluten content;
• Dataset 5: storage time (day), atmospheric temperature, and protein content.

This organization of the experimental data allows separate empirical modeling of moisture and temperature evolution, as well as quality-related parameters during storage. Furthermore, the division into available observations facilitates the application of mathematical models specifically adapted to each type of analyzed phenomenon, thereby ensuring the consistency, reliability, and relevance of the modeling results.

The selected datasets were constructed to include both storage time and environmental variables, thereby enabling the mathematical models to describe quality evolution as a function of temporal progression and climatic conditions simultaneously. This approach was adopted because wheat quality changes during storage are governed by the combined influence of these factors under real storage conditions.

The selection of atmospheric temperature as an independent variable in Datasets 3–5 is justified from both physical and methodological perspectives. Under static grain storage conditions, without forced aeration, atmospheric temperature represents the dominant external factor governing the thermal regime of the stored grain mass and indirectly influencing all physical and biochemical processes occurring within it.

From a thermodynamic perspective, product temperature cannot be considered a truly independent variable, as it responds with a certain degree of thermal inertia to fluctuations in atmospheric temperature. The latter generates the thermal gradients responsible for moisture redistribution and for the intensity of metabolic processes occurring within the grain bulk.

At the same time, wheat quality parameters such as test weight, gluten content, and protein content evolve as a result of temperature-dependent kinetic processes and are directly associated with the intensity of biochemical reactions as well as enzymatic and microbiological activity. Consequently, atmospheric temperature constitutes a primary driving factor affecting the rate of quality deterioration during storage.

From the standpoint of three-dimensional mathematical modeling, atmospheric temperature satisfies the requirements of a genuine independent variable. It is determined by factors external to the storage system, can be measured directly, and is not influenced by the quality parameters under investigation. This characteristic reduces the risk of multicollinearity and contributes to the robustness and stability of the resulting mathematical models.

Furthermore, the broad and continuous variation of atmospheric temperature allows the generation of mathematically stable and physically interpretable response surfaces, compatible with the requirements of TableCurve 3D applications. In this context, the use of atmospheric temperature as an independent variable in the datasets dedicated to quality parameter analysis ensures both the conceptual consistency of the study and a direct relationship between mathematical modeling and the actual dynamics of quality degradation processes occurring during storage.

To ensure a coherent interpretation of the investigated phenomena and to facilitate the identification of representative mathematical models, the five datasets were grouped into two major categories according to the nature of the processes under study.

The first category, referred to as the transfer model (physical model), comprises the datasets related to moisture and temperature and aims to describe the mechanisms of mass and energy transfer between the surrounding environment and the stored grain bulk.

The second category, defined as the degradation model (quality model), includes the datasets associated with test weight, gluten content, and protein content and is intended to characterize the quality deterioration processes occurring during storage.

This classification enables a differentiated analysis of the investigated phenomena and provides the necessary framework for identifying mathematical models specifically adapted to each process type, taking into account their distinct physical and biochemical characteristics.

### 4.1. Development of Empirical Predictive Models for Moisture and Temperature Dynamics During Storage

To identify a common mathematical model for the moisture and temperature transfer processes represented by Datasets 1 and 2, the TableCurve 3D software package was employed. This application automatically generates a large number of candidate equations based on the experimental data provided. A total of 463 mathematical relationships were generated for Dataset 1, whereas 372 relationships were generated for Dataset 2. The analysis of these equations focused on identifying functionally similar forms with physical significance, capable of supporting the development of a common mathematical model that could consistently describe the hygrothermal transfer processes occurring within the stored grain mass during storage.

To identify a set of common equations representative of the analyzed datasets, the model selection and validation methodology described in the literature [46,47] was applied. This approach involves the progressive filtering of the generated equations according to both mathematical criteria and physical relevance (Figure 6). By applying this methodology, 193 common equations were identified among the models generated by TableCurve 3D, together with 270 equations unique to Dataset 1 and 179 equations unique to Dataset 2.

Further selection was performed by imposing a coefficient of determination threshold of R^2^ ≥ 0.80, which reduced the number of common equations to 51. This substantially narrowed the selection domain and enabled the identification of an optimal set of candidate models. Among these, nine equations corresponding to specific entries in the TableCurve 3D equation database were retained for further evaluation. The remaining common equations were excluded from the analysis due to their high mathematical complexity, being formulated as Chebyshev series (including logarithmic variants), Fourier series (univariate or bivariate), bivariate cosine series, or sigmoidal functions. Such formulations are generally more difficult to implement in engineering applications and offer limited advantages with respect to the physical interpretation of the phenomena under investigation.

Equation (1), corresponding to Equation No. 64 in the TableCurve 3D database, is presented below. This equation was selected from the final set of nine candidate relationships following a comprehensive evaluation procedure that included both the visual assessment of the generated three-dimensional response surfaces (Figure 7) and the analysis of the magnitude and statistical significance of the equation coefficients.

(1) z = a + bx + cx^2^ + dx^3^ + ex^4^ + fx^5^ + gy + hy^2^ + iy^3^ + jy^4^

The selection process was aimed at identifying the model that provided the best agreement between the mathematical representation and the underlying physical phenomenon while simultaneously achieving an optimal balance between statistical accuracy and engineering interpretability.

Table 2 presents the values of the coefficients associated with Equation (1), together with the corresponding coefficients of determination (R^2^) obtained for each analyzed dataset.

To evaluate the goodness-of-fit of the proposed mathematical model, the residuals associated with the fitted equations were analyzed and graphically represented (Figure 8). Residual plots were included to illustrate the distribution of errors between the experimental observations and the values predicted by the model, thereby providing a means of assessing the adequacy of the selected mathematical function.

The analysis of these graphical representations provides valuable information regarding the presence of potential systematic deviations, nonlinear trends not captured by the model, or the occurrence of outlying observations. Consequently, residual analysis contributes to verifying both the statistical validity and the physical consistency of the identified mathematical relationship.

### 4.2. Identification of the Mathematical Model Describing the Quality Degradation Processes of the Stored Product

This subsection focuses on the identification of mathematical models associated with the degradation processes of the main quality parameters of stored wheat, namely test weight, gluten content, and protein content. These processes exhibit a complex and nonlinear nature, resulting from the interaction between environmental factors and the internal biochemical mechanisms of the product, whose intensity varies throughout the storage period.

Unlike the physical transfer processes discussed previously, quality degradation is directly influenced by climatic conditions, particularly atmospheric temperature, which governs the rate of internal reactions and the evolution of the physicochemical properties of the stored product. In this context, the adopted modeling methodology aims to identify three-dimensional mathematical relationships capable of describing the dependencies among storage time, environmental factors, and quality parameters based on the corresponding experimental datasets. The model selection procedure considers both statistical validation criteria and the physical interpretability of the obtained relationships, with the objective of highlighting the mechanisms governing the long-term deterioration of product quality.

Similar mathematical approaches have been applied in the study of complex dynamic systems, where the influence of geometric parameters on the motion characteristics of jaw crusher mechanisms was evaluated through mathematical and numerical analyses, highlighting the effectiveness of modeling techniques for predicting process behavior [55]. Furthermore, alternative mathematical formulations have been proposed for the kinematic analysis of jaw crusher drive mechanisms, demonstrating the importance of selecting appropriate mathematical functions to accurately describe nonlinear engineering processes and system dynamics [56].

To identify a mathematical model representative of the quality degradation processes corresponding to Datasets 3, 4, and 5, the TableCurve 3D software was employed. The application generated 472 equations for Dataset 3, 547 equations for Dataset 4, and 552 equations for Dataset 5 (Figure 9). By applying the common-equation identification methodology, 263 common equations were identified, together with 209, 284, and 289 equations unique to Datasets 3, 4, and 5, respectively.

Subsequently, by imposing a stringent coefficient of determination threshold of R^2^ ≥ 0.90, the number of common equations was reduced to 99, thereby narrowing the selection domain to an optimal subset of candidate models. The selection criteria were similar to those employed in Section 4.1 and were based on achieving a balance between statistical performance, reduced mathematical complexity, and physical interpretability. Consequently, excessively complex relationships that did not provide significant additional benefits in describing the investigated phenomena were excluded from further analysis.

**Figure 10:**
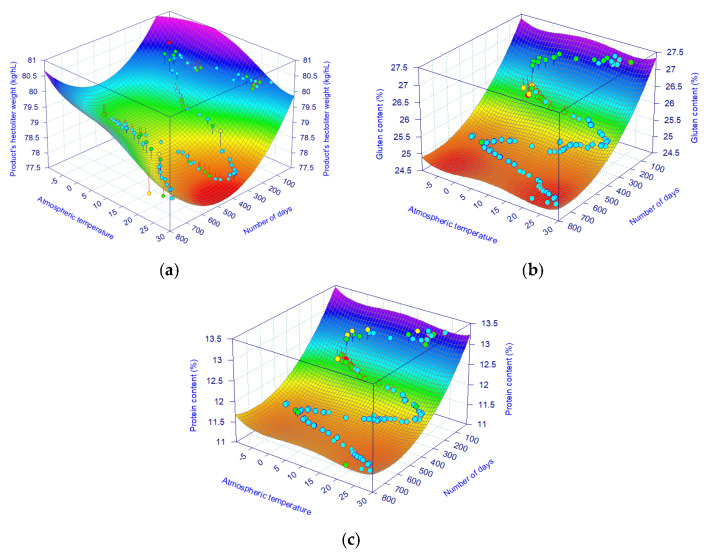
Three-dimensional response surfaces obtained for: (**a**) Dataset 3 (test weight); (**b**) Dataset 4 (gluten content); and (**c**) Dataset 5 (protein content).

The equation selected from the final set of 28 candidate equations is presented below. The corresponding response surfaces generated by this mathematical relationship are illustrated in Figure 10 for each of the analyzed quality parameters.

(2) z = a + bx + cx^2^ + dy + ey^2^ + fy^3^ + gy^4^

The coefficients associated with Equation (2), along with the corresponding coefficients of determination (R^2^), are presented in Table 3 for each dataset analyzed.

**Figure 11:**
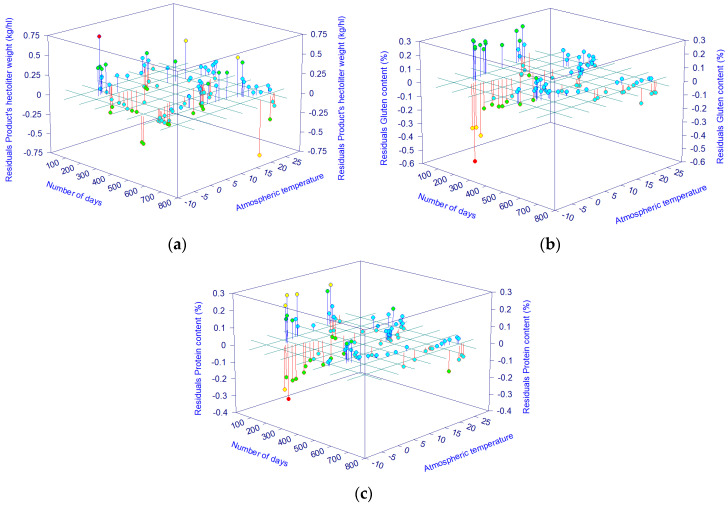
Residual plots for: (**a**) Dataset 3; (**b**) Dataset 4; (**c**) Dataset 5.

To evaluate the goodness-of-fit of the proposed mathematical model, the residuals associated with the fitted equations were analyzed and graphically represented (Figure 11). Residual analysis provides important information regarding the distribution of deviations between the experimental observations and the values predicted by the model, thereby contributing to the assessment of model adequacy and predictive performance.

In addition to residual analysis, model performance was subsequently evaluated using a temporal validation dataset corresponding to the 2024–2025 storage period. The validation procedure and the associated statistical performance indicators (MSE, RMSE, MAE, MAPE, and NSE) are presented in Section 4.3.

The adequacy of the selected mathematical models was evaluated using coefficients of determination, residual analysis, and additional statistical performance indicators presented in Section 4.3.

### 4.3. Evaluation of Mathematical Model Performance

The identification of a mathematical model with a high coefficient of determination (R^2^) represents only the first stage of the modeling process. In addition to goodness-of-fit indicators, the adequacy of the selected equations was assessed through residual analysis and by evaluating multiple statistical performance indicators calculated using the available experimental observations [57].

In the present study, model performance was evaluated by comparing the values predicted by the identified mathematical equations with the corresponding experimental observations. This approach enabled the quantification of prediction errors and the assessment of the capability of the selected equations to reproduce the observed behavior of the monitored parameters.

The model evaluation process was aimed at quantifying the differences between the experimental observations and the values predicted by the proposed mathematical models, as well as evaluating their predictive performance. To ensure a comprehensive and objective assessment, both absolute and squared error metrics, as well as percentage-based indicators and efficiency coefficients commonly used in predictive model validation, were employed.

Accordingly, model validation was performed using the following statistical indicators:

• MSE (Mean Squared Error) used to evaluate the average squared prediction error;
• RMSE (Root Mean Squared Error) used to express the average prediction error in the units of the analyzed parameter;
• MAE (Mean Absolute Error) used to determine the mean absolute prediction error;
• MAPE (Mean Absolute Percentage Error) used to evaluate the average percentage error and assess prediction accuracy;
• NSE (Nash–Sutcliffe Efficiency Coefficient) used to evaluate the overall performance of the model and compare it with the mean of the observed experimental values.

The simultaneous use of these indicators allows model performance to be assessed from complementary perspectives. While MSE and RMSE are sensitive to large-magnitude errors, MAE provides a direct and easily interpretable measure of the average deviations between observed and predicted values. In contrast, MAPE expresses the relative error in percentage terms, thereby facilitating the evaluation of prediction accuracy. The Nash–Sutcliffe efficiency coefficient complements the analysis by quantifying the ability of the model to reproduce the variability observed in the experimental data.

The application of this validation framework was intended to verify the degree of agreement between the experimental observations and the model predictions, as well as to demonstrate the capability of the developed mathematical models to describe and predict the evolution of the physical and quality-related parameters of wheat during long-term storage.

The results of the mathematical model validation are presented in Table 4. For each analyzed parameter, the MSE, RMSE, MAE, MAPE, and NSE indicators were calculated. For the temperature model, the MAPE indicator was not computed because the dataset contains values close to zero as well as negative values, conditions under which percentage error metrics may produce unstable and difficult-to-interpret results.

For the temperature parameter, the MAPE (Mean Absolute Percentage Error) metric was not calculated because the dataset contains values close to zero as well as negative values. Under such conditions, the use of MAPE may lead to distorted and difficult-to-interpret results, since the percentage error is computed relative to the observed value. When the actual values approach zero, even small deviations can produce excessively large percentage errors, whereas for negative values, the physical meaning of the percentage error becomes questionable.

For this reason, the performance of the temperature model was evaluated using the MSE, RMSE, MAE, and NSE metrics, which are generally considered more robust and appropriate for datasets containing negative values or observations close to zero.

The statistical validation results demonstrate the high predictive capability of the mathematical models developed to describe the evolution of the main wheat quality parameters during storage.

The model developed for moisture prediction exhibited good predictive performance, with a coefficient of determination of R^2^ = 0.82, indicating that 82% of the variability in the experimental data is explained by the model. The low error values (MSE = 0.05, RMSE = 0.24 and MAE = 0.18) suggest only minor deviations between the predicted and experimentally measured values. Furthermore, the MAPE value of 1.42% indicates a very high level of prediction accuracy. The Nash–Sutcliffe efficiency coefficient (NSE = 0.99) confirms the excellent performance of the model and its ability to accurately reproduce the experimentally observed moisture variations. Although the moisture model exhibited the lowest coefficient of determination among the analyzed parameters, it nevertheless maintained a very satisfactory predictive performance.

The model developed for temperature prediction demonstrated very good predictive performance, as reflected by the high coefficient of determination (R^2^ = 0.95) and the Nash–Sutcliffe efficiency coefficient (NSE = 0.95), both indicating excellent agreement between the predicted and experimental values. The error indicators (MSE = 5.22, RMSE = 2.28 °C, and MAE = 1.74 °C) show that the prediction errors are relatively small compared with the overall range of temperature variations observed during the experiment. For this parameter, the MAPE metric was not calculated because the dataset contains values close to zero and/or negative values, under which conditions the interpretation of percentage-based errors may become unreliable or misleading.

The results obtained for hectoliter weight (MSE = 0.05, RMSE = 0.23, MAE = 0.18, and MAPE = 0.239%) demonstrate the very high accuracy of the developed model. The exceptionally high Nash–Sutcliffe Efficiency coefficient (NSE = 0.999) confirms the model’s excellent capability to reproduce the experimental variability of this parameter, with differences between measured and predicted values being practically negligible.

For gluten content, the mathematical model produced highly accurate predictions, as reflected by the low error values (RMSE = 0.14, MAE = 0.104, and MAPE = 0.403%). Furthermore, the NSE coefficient of 0.999 indicates an almost perfect agreement between experimental and estimated values, confirming the good agreement between observed and predicted values within the investigated dataset.

The best predictive performance was achieved for protein content, for which the lowest statistical error values were recorded (MSE = 0.01, RMSE = 0.10, and MAE = 0.07), together with an MAPE value of only 0.56%. The very high NSE coefficient (0.998), combined with an R^2^ value of 0.97, indicates that the model reproduces the experimental behavior of the analyzed parameter with remarkable accuracy.

Overall, all validated models exhibited low error indicators and NSE coefficients ranging from 0.95 to 0.999, demonstrating their ability to accurately describe and predict the evolution of the analyzed parameters throughout the storage period. MAPE values below 2% for all models for which this indicator was applicable further confirm the excellent accuracy of the predictions. Therefore, the developed mathematical models demonstrated satisfactory predictive performance within the investigated dataset and may serve as useful tools for predicting changes in the physical and quality properties of wheat under conditions similar to those investigated in the present study.

## 5. Discussion

The results obtained in the present study indicate an association between the effects of climatic variability and wheat quality evolution on the evolution of quality parameters of wheat stored over long periods, even under conditions where the same storage infrastructure, storage technology, and wheat type were used. This experimental approach reduced the influence of technological variability by using the same storage facility, wheat cultivar, and storage protocol across both years. Nevertheless, because only two storage periods were evaluated, the observed differences should be interpreted as climate-associated responses rather than unequivocal evidence of causal climatic effects. The findings confirm observations reported in the literature, according to which temperature and humidity are the dominant external factors governing the stability of stored wheat and the rate of deterioration of its technological characteristics.

An important limitation of the present study should be acknowledged. The investigation was performed on a single wheat lot monitored continuously during two consecutive years of storage. Consequently, the observed differences between the 2023–2024 and 2024–2025 periods may reflect not only differences in environmental conditions but also the effects associated with increasing storage duration. Therefore, the individual contributions of climatic variability and storage aging cannot be fully separated within the framework of the present experimental design.

A comparative analysis of the two storage periods revealed that the 2024–2025 storage cycle exhibited slightly higher thermal variability, as reflected by an increase in atmospheric thermal amplitude from approximately 33 °C to 34 °C and in grain thermal amplitude from approximately 30 °C to 32 °C. The increased thermal amplitude observed in the wheat mass may reflect a more pronounced influence of external climatic conditions on the internal thermal regime of the storage facility. Although the grain mass exhibited lower thermal amplitudes than the surrounding environment, the present study did not directly quantify thermal inertia through lag-time or cross-correlation analyses. Therefore, the observed buffering effect should be interpreted as an empirical observation derived from the monitored temperature dynamics. Consequently, the observed quality changes may be associated with the combined influence of storage duration, moisture exchange processes, and temperature-dependent biochemical reactions during the 2024–2025 period. However, these mechanisms were not directly measured and therefore should be regarded as plausible interpretations of the observed trends.

The moisture content of the wheat showed relatively small variations throughout the two experimental cycles; however, the differences observed between annual mean values indicate the existence of a continuous hygroscopic equilibrium process between the grain bulk and the storage atmosphere. The slight increase in average wheat moisture content during the 2024–2025 period may be associated with the combined influence of environmental conditions and long-term moisture equilibration processes occurring within the stored grain mass. Although the atmospheric relative humidity patterns were very similar during the two storage periods, small cumulative differences may have contributed to the observed moisture evolution. Although temperature-dependent metabolic and biochemical reactions may have contributed to quality changes, these processes were not directly quantified in the present study. Although the percentage changes were relatively small, the results demonstrate that apparently minor modifications in the hygrometric regime can generate significant cumulative effects during long-term storage.

The evolution of hectoliter weight confirms the existence of a slow but continuous deterioration process affecting the physical characteristics of the stored product. The decrease in mean values and the increase in variability observed during the 2024–2025 storage period indicate a high sensitivity of this parameter to climatic conditions. This phenomenon may be associated with storage-related physicochemical changes reported in the literature. However, because respiration rate, dry matter loss, and structural modifications were not directly measured in the present study, the underlying mechanisms cannot be conclusively identified. Although the recorded values remained within technologically acceptable limits, the downward trend emphasizes the need for continuous monitoring of this parameter during extended storage periods.

Particular attention should be paid to the evolution of protein-related parameters, namely gluten and protein contents, as these directly influence the bread-making quality of wheat. The obtained results show that both indicators exhibited more pronounced reductions during the 2024–2025 storage period compared to the previous year. The increased amplitude of variation and the decline in mean values suggest that higher temperatures and a more variable hygrometric regime may have contributed to the observed decline of protein-related quality parameters. These findings are consistent with numerous studies reporting that elevated temperatures accelerate structural modifications of gluten proteins and consequently reduce the technological quality of flour.

Several mechanisms may explain the observed decrease in protein content under conditions of increased climatic variability. Temperature fluctuations can intensify grain respiration and metabolic activity during storage, leading to the consumption of reserve substances and accelerating biochemical transformations within the grain. In addition, repeated moisture adsorption and desorption cycles associated with changes in environmental relative humidity may affect the stability of protein structures and promote enzymatic reactions involved in protein degradation. Although the present study did not directly investigate the biochemical pathways responsible for these changes, the more pronounced reductions in protein content observed during the 2024–2025 storage period are consistent with the higher thermal variability recorded during that year. Similar observations have been reported in previous studies, which indicate that elevated storage temperatures and fluctuating moisture conditions may contribute to reductions in protein quality and technological performance of wheat during long-term storage [21,22].

The comparison of quality parameters revealed the existence of an interdependent relationship among hectoliter weight, protein content, and gluten content. The simultaneous decline of these indicators suggests that the processes responsible for quality deterioration do not act independently but rather constitute a complex mechanism governed by storage conditions and the biological response of the grain mass. The persistence of these correlations throughout both study years demonstrates the existence of stable mechanisms that regulate wheat quality evolution during storage, regardless of the degree of climatic variability.

An important aspect of this research was the development and validation of mathematical models designed to describe transfer and degradation phenomena occurring during storage. The high coefficients of determination (R^2^ = 0.82–0.97), together with the excellent Nash–Sutcliffe efficiency values (NSE = 0.95–0.99), demonstrate the capability of the proposed models to accurately reproduce the experimental behavior of the analyzed parameters. In particular, the models developed for hectoliter weight, gluten content, and protein content exhibited outstanding predictive performance, confirming that temperature and storage duration are highly relevant explanatory variables for quality degradation processes.

A potential limitation of the proposed modeling approach is the possibility of partial confounding between storage duration and environmental variables, since both factors evolve simultaneously during the storage process. To reduce this effect, the mathematical models were formulated using storage time together with independently measured environmental parameters (atmospheric temperature or relative humidity), allowing both temporal and climatic components to contribute separately to the model response. Nevertheless, because wheat quality evolution results from the combined action of storage duration and environmental conditions, the developed models should be interpreted as empirical predictive relationships describing their joint influence rather than as tools for quantifying the isolated contribution of each individual factor.

The performance indicators were calculated by comparing experimental observations with the values predicted by the identified mathematical models. The low statistical error values and the reduced MAPE values obtained for the quality parameters indicate that the models can serve as valuable tools for forecasting product evolution and establishing critical intervention thresholds in grain storage facilities. From a practical perspective, the integration of these models into digital monitoring systems could contribute to the development of proactive inventory management strategies.

These findings are consistent with recent studies demonstrating that intelligent prediction systems integrating temperature, moisture, and quality indicators can accurately model wheat quality evolution under different storage conditions. Advanced machine-learning frameworks have shown considerable potential for supporting grain storage management by improving the prediction and evaluation of quality changes during long-term storage [24].

For this reason, the observed differences should be interpreted as the combined response of the stored grain to both storage duration and year-to-year variations in environmental conditions. Although climatic variability appears to be associated with changes in quality evolution, the current dataset does not allow a complete separation of climatic effects from those resulting from prolonged storage.

Overall, the results indicate that interannual climatic variability is associated with differences in mass and energy transfer processes, as well as in wheat quality evolution during storage. However, because the same grain lot was monitored continuously throughout the study period, the observed effects should be interpreted together with the influence of storage duration. Even under controlled and continuously monitored storage conditions, external climatic changes are transmitted to the grain bulk and affect its long-term stability. These findings highlight the need for adaptive storage strategies based on continuous monitoring, predictive modeling, and technological interventions tailored to the specific climatic conditions of each storage year. Furthermore, the study contributes to a better understanding of the mechanisms governing wheat preservation and provides a solid scientific basis for optimizing post-harvest management practices in the context of increasing climate variability.

## 6. Conclusions

The comparative analysis indicates that differences in climatic conditions between the two storage periods were associated with measurable differences in wheat quality evolution. Because the same grain lot was monitored continuously throughout the two-year storage period, the observed differences between the 2023–2024 and 2024–2025 intervals should be interpreted as the combined effect of storage duration and environmental variability rather than as definitive evidence of isolated climatic effects. Consequently, the present findings describe climate-associated responses observed during long-term storage under the investigated conditions. The experimental findings revealed that wheat moisture content remained closely related to the relative humidity of the storage environment, reflecting the continuous hygroscopic equilibrium established between the grain mass and the surrounding atmosphere. At the same time, grain temperature followed the fluctuations of ambient temperature, although with attenuated variations due to the thermal inertia characteristic of bulk cereal products. However, the slightly higher thermal amplitudes observed during the 2024–2025 storage period may reflect a somewhat greater influence of environmental conditions on the stored grain mass, although the contribution of storage duration cannot be completely separated within the framework of the present study.

The results further showed that hectoliter weight progressively decreased throughout storage. This decline represents an observed quality trend during prolonged storage and may be associated with physicochemical changes occurring within the grain mass, although the underlying mechanisms were not directly investigated in the present study. This reduction appeared more pronounced during the storage period characterized by greater climatic variability and may reflect the sensitivity of this quality parameter to environmental conditions. Similarly, both gluten and protein contents exhibited a continuous declining trend, with a more substantial deterioration observed during the 2024–2025 period. These findings are consistent with the hypothesis that protein fractions are sensitive to fluctuations in temperature and humidity and highlight the importance of maintaining stable storage conditions in order to preserve the technological quality of wheat intended for processing.

Because formal statistical significance testing was not performed, the observed differences between the two storage periods should be interpreted as descriptive comparisons rather than statistically confirmed effects.

The simultaneous evolution of hectoliter weight, protein content, and gluten content during storage suggests the existence of common deterioration trends affecting these quality parameters. Similar temporal patterns were observed throughout both storage periods, indicating that these indicators responded in a comparable manner to the storage conditions investigated. However, no formal statistical correlation analysis was performed in the present study; therefore, these observations should be interpreted descriptively rather than as statistically demonstrated relationships among the analyzed parameters.

A major outcome of the study was the successful development and performance evaluation of mathematical models capable of reproducing the observed evolution of moisture, temperature, and wheat quality parameters during storage. The transfer model showed high coefficients of determination (R^2^ = 0.82 for moisture and R^2^ = 0.95 for temperature), together with favorable statistical performance indicators, including NSE values of 0.99 and 0.95, respectively. Likewise, the models developed for quality parameters such as hectoliter weight, gluten, and protein content exhibited agreement with the experimental observations, with R^2^ values ranging from 0.90 to 0.97 and NSE values between 0.99 and 0.99.

The mathematical models were developed using the available experimental observations collected during the monitored storage periods. Their adequacy was assessed through coefficients of determination, residual analysis, and statistical performance indicators. The strong agreement between predicted and experimental values indicates that the selected mathematical relationships provide a satisfactory representation of the observed behavior under the investigated storage conditions. In addition, because the dataset was obtained from a single grain lot monitored over two consecutive storage years, the developed models describe the combined influence of storage duration and environmental conditions. Therefore, they should be regarded as empirical predictive tools applicable to conditions similar to those investigated in the present study. However, because the models were evaluated using data obtained from a single storage facility and one wheat cultivar, their broader applicability should be interpreted with caution. Further evaluation using additional grain lots, storage facilities, and climatic conditions would be required before broader applicability can be established. Nevertheless, the favorable model performance indicators highlight the potential usefulness of these models as tools for supporting cereal storage management.

The proposed equations should therefore be regarded as empirical predictive tools developed from experimental observations under the investigated storage conditions and not as mechanistic models describing the fundamental physical or biochemical processes occurring within the grain mass. Nevertheless, the favorable model performance indicators highlight the potential usefulness of these models as tools for supporting cereal storage management.

Overall, the research demonstrates that the integration of climatic monitoring and mathematical modeling can contribute to the description of wheat quality evolution during long-term storage under the investigated conditions. The findings highlight the potential value of incorporating climatic information into grain storage management, particularly in the context of increasing climatic variability and quality preservation requirements.

The results suggest that continuous monitoring of temperature and moisture, together with predictive modeling approaches, may support the identification of periods associated with increased quality deterioration. Although the present study did not directly evaluate storage intervention strategies or decision-support systems, the developed models may provide a useful basis for future investigations aimed at improving post-harvest management practices under varying climatic conditions.

## Figures and Tables

**Table 1:** Initial physicochemical characteristics of the wheat used in the study.

Parameter	Unit	Value
Initial moisture content	%	13.1
Initial grain temperature	°C	24
Test weight	kg hL^−1^	80.1
Wet gluten content	%	27.1
Protein content	%	13.1

**Table 2:** Coefficients of the mathematical model and corresponding coefficients of determination (R^2^) for Datasets 1 and 2.

No.	Dataset			a	b	c	d	e	f	g	h	i	j
1	Dataset 1	R^2^ Value	0.82	15.0266620570297	−0.008929691235273360	0.0000933482288210984	−0.000000414574329722878	0.000000000752012564484504	−0.000000000000464452391160192	−0.142382911350046	0.00244163411460561	−0.0000093393160027288	−0.0000000164653372832365
		Standard Error		5.335274	0.006232	5.38 × 10^−5^	1.83 × 10^−7^	2.68 × 10^−10^	1.41 × 10^−13^	0.365763	0.00909	9.76 × 10^−5^	3.83 × 10^−7^
		T Value		2.816474	−1.43282	1.734833	−2.26251	2.807239	−3.29955	−0.38928	0.2686	−0.0957	−0.04304
		95% CI Lower		4.420488	−0.02132	−1.4 × 10^−5^	−7.8 × 10^−7^	2.19 × 10^−10^	−7.4 × 10^−13^	−0.8695	−0.01563	−0.0002	−7.8 × 10^−7^
		IC 95% superior		25.63284	0.00346	0.0002	−5 × 10^−8^	1.28 × 10^−9^	−1.8 × 10^−13^	0.58473	0.020512	0.000185	7.44 × 10^−7^
		Approx. *p*-value		0.0049	0.1519	0.0828	0.0237	0.005	0.001	0.6971	0.7882	0.9238	0.9657
2	Dataset 2	R^2^ Value	0.95	2.24072718411271	−0.129872855402828	0.00152427368594123	−0.00000633514983695217	0.0000000105541829813025	−0.00000000000605327696313269	0.7764222237383060	0.00844918780976915	0.0000910344776976163	−0.00000069417023102642
		Standard Error		2.167701805	0.054523185	0.000444011	1.45619 × 10^−6^	2.08086 × 10^−9^	1.08187 × 10^−12^	0.123987909	0.024046993	0.001975234	4.46913 × 10^−5^
		T Value		1.033687927	−2.381974857	3.432966691	−4.350510647	5.072032981	−5.595212768	6.262080126	0.351361515	0.046087952	−0.015532566
		95% CI Lower		−2.068521384	−0.23826136	0.00064161	−9.22995 × 10^−6^	6.41757 × 10^−9^	−8.20396 × 10^−12^	0.529942417	−0.039354652	−0.0038356	−8.95375 × 10^−5^
		95% CI Upper		6.549975752	−0.021484351	0.002406938	−3.44035 × 10^−6^	1.46908 × 10^−8^	−3.9026 × 10^−12^	1.02290203	0.056253027	0.004017669	8.81492 × 10^−5^
		Approx. *p*-value		0.3013	0.0172	0.0006	0.000014	<0.000001	<0.000001	<0.000001	0.7253	0.9632	0.9876

**Table 3:** Model coefficients and coefficients of determination (R^2^) corresponding to the quality degradation model for Datasets 3, 4, and 5.

No.	Dataset			a	b	c	d	e	f	g
1	Dataset 3	R^2^ Value	0.9	81.0989031288991	−0.00959371869092624	0.0000102294457010306	−0.0254894833159521	0.00159617102435664	−0.000221842513900753	0.00000503283785197374
		Standard Error		0.087926	0.000493	6.56 × 10^−7^	0.012377	0.002372	0.000195	4.42 × 10^−6^
		T Value		922.3571	−19.4537	15.60336	−2.0595	0.672947	−1.13995	1.13935
		95% CI Lower		80.9242	−0.01057	8.93 × 10^−6^	−0.05008	−0.00312	−0.00061	−3.7 × 10^−6^
		95% CI Upper		81.27361	−0.00861	1.15 × 10^−5^	−0.0009	0.006309	0.000165	1.38 × 10^−5^
		Approx. *p*-value		<0.000001	<0.000001	<0.000001	0.0395	0.5010	0.2543	0.2546
2	Dataset 4	R^2^ Value	0.96	27.2121397876678	−0.00697967008484462	0.00000486089122836979	0.0130392107841012	0.000973542015815864	−0.000155954204553037	0.0000037677178428154
		Standard Error		0.054331	0.000305	4.05 × 10^−7^	0.007648	0.001466	0.00012	2.73 × 10^−6^
		T Value		500.8576	−22.9043	11.99912	1.704978	0.664238	−1.2969	1.380352
		95% CI Lower		27.10419	−0.00759	4.06 × 10^−6^	−0.00216	−0.00194	−0.00039	−1.7 × 10^−6^
		95% CI Upper		27.32009	−0.00637	5.67 × 10^−6^	0.028235	0.003886	8.3 × 10^−5^	9.19 × 10^−6^
		Approx. *p*-value		<0.000001	<0.000001	<0.000001	0.0882	0.5065	0.1947	0.1675
3	Dataset 5	R^2^ Value	0.97	13.2129475262491	−0.00576534940623673	0.00000442158975020488	0.00271538376298434	0.00113464301838699	−0.000123478924933295	0.00000275925931952683
		Standard Error		0.037827	0.000212	2.82 × 10^−7^	0.005325	0.00102	8.37 × 10^−5^	1.9 × 10^−6^
		T Value		349.3024	−27.1743	15.67698	0.509975	1.111933	−1.47486	1.451959
		95% CI Lower		13.13779	−0.00619	3.86 × 10^−6^	−0.00786	−0.00089	−0.00029	−1 × 10^−6^
		95% CI Upper		13.28811	−0.00534	4.98 × 10^−6^	0.013295	0.003162	4.29 × 10^−5^	6.54 × 10^−6^
		Approx. *p*-value		<0.000001	<0.000001	<0.000001	0.6101	0.2662	0.1403	0.1465

**Table 4:** Values of the statistical indicators used for assessing the performance of the mathematical models developed for the analyzed parameters.

Parameter	R^2^	MSE	RMSE	MAPE (%)	NSE
Moisture	0.82	0.05	0.24	1.42	0.99
Temperature	0.95	5.22	2.28	-	0.95
Test Weight	0.9	0.05	0.23	0.24	0.99
Gluten Content	0.95	0.02	0.14	0.40	0.99
Protein Content	0.97	0.01	0.1	0.56	0.99

## Data Availability

The original contributions presented in this study are included in the article/Supplementary Materials. Further inquiries can be directed to the corresponding authors.

## Supplementary Materials

The following supporting information can be downloaded at: https://www.mdpi.com/article/10.3390/foods15152726/s1.
